# The role of chronic obstructive pulmonary disease mediated by immune cells on lung adenocarcinoma: A two-step, two-sample Mendelian randomization study

**DOI:** 10.1097/MD.0000000000045248

**Published:** 2025-11-14

**Authors:** Xinyu Liu, Jing Xu, Sheng Wang

**Affiliations:** a Respiratory Department, Zhejiang Jinhua Guangfu Cancer Hospital, Jinhua, Zhejiang, China; b Pharmacy Department, Jinhua People’s Hospital, Jinhua, Zhejiang, China; c Science and Education Department, Zhejiang Jinhua Guangfu Cancer Hospital, Jinhua, Zhejiang, China.

**Keywords:** chronic obstructive pulmonary disease, lung adenocarcinoma, Mendelian randomization, Treg

## Abstract

Chronic obstructive pulmonary disease (COPD) commonly co-occurs with lung cancer, particularly lung adenocarcinoma (LUAD), suggesting a potential shared molecular mechanism and risk factors between the 2 conditions. This study aimed to explore the causal relationship between COPD and LUAD mediated by immune cells using a 2-step, 2-sample Mendelian randomization (MR) analysis. The random-effect inverse variance weighted method, which combines the Wald ratio of individual single-nucleotide polymorphisms, was employed as the primary approach for causal inference, with random-effects models utilized in the presence of heterogeneity. Mediation analysis was conducted to assess indirect effects in the pathway from COPD to LUAD. The MR analysis demonstrated that COPD increased the risk of LUAD (odds ratio = 1.180, 95% confidence interval [CI]: 1.004–1.387, *P* = .045). Furthermore, among 40 immune cell traits examined, 5 were associated with an elevated risk of LUAD, while 6 exhibited a detrimental effect. Importantly, the mediation MR analysis revealed that the indirect impact of COPD on LUAD was partially mediated by Activated & resting Treg cells (mediation effect: 0.010, 95% CI: 0.001–0.021; *P* = .047) and Activated & secreting Treg cells (mediation effect: 0.004, 95% CI: 0.001–0.008; *P* = .044). These findings suggest a positive association between COPD and LUAD, with a partial mediation effect through Activated & resting Treg cells and Activated & secreting Treg cells.

## 1. Introduction

The World Health Organization’s projection that chronic obstructive pulmonary disease (COPD), the prevailing chronic lung ailment, would rank as the third leading cause of global mortality by 203 has materialized earlier than anticipated.^[1,2]^ In 2019, there were over 200 million documented cases of COPD, resulting in a mortality toll exceeding 3.3 million individuals worldwide.^[1,2]^

COPD patients are at a significantly higher risk of developing lung cancer (LC) compared to non-COPD patients, with a 4- to 6-fold increased likelihood.^[3,4]^ The 1-year incidence of LC in COPD patients ranges from 8% to 1.7%, whereas in non-COPD patients, it is only 2%. Furthermore, the 10-year risk of LC is 8.8% for COPD patients and 2% for those without COPD.^[5,6]^ These statistics suggest a potential shared molecular mechanism and risk factor between LC and COPD. However, further investigation is required to elucidate the interactions and underlying mechanisms linking these two diseases. Therefore, exploring the mechanisms of COPD and LC is a valuable area of research.

COPD is characterized by persistent airway obstruction and inflammation in the lower respiratory tract, resulting in a gradual and irreversible decline in lung function.^[7]^ The infiltration of various inflammation and immune cells, including neutrophils, macrophages, T cells, and B cells, into the central airway, distal airway, and lung parenchyma is considered the primary pathogenic mechanism of COPD.^[8]^ The progression to LC in COPD involves a complex multistep process in which the immune system plays a pivotal role. Immune cells are responsible for identifying and eliminating cancerous cells through immune surveillance. However, tumors can evade detection by the immune system and promote their growth by inhibiting the recognition of tumor-associated antigens.^[9,10]^ Consequently, immune dysfunction is believed to be a critical factor in the progression from COPD to LC. Specifically, in individuals with COPD, the increased infiltration of neutrophils and macrophages exacerbates emphysema and supports cancer development by releasing immune factors and metalloproteinases.^[8]^

Mendelian randomization (MR) analysis, a robust statistical approach rooted in genome-wide association studies, serves to elucidate causal links between exposures and outcomes in epidemiology.^[11]^ By leveraging single-nucleotide polymorphisms (SNPs) as instrumental variables (IVs), MR analysis enables the exploration of causal relationships with reduced susceptibility to confounding factors and reverse causation, akin to a natural randomized controlled trial.^[12]^ Lung adenocarcinoma (LUAD) is the predominant subtype of LC, comprising over 40% of all cases. In this study, we conducted a comprehensive 2-sample 2-step MR analysis to assess the impact of COPD on LUAD and investigate the potential mediating role of immune cells.

## 2. Materials and methods

### 2.1. Study design

**Figure 1.**
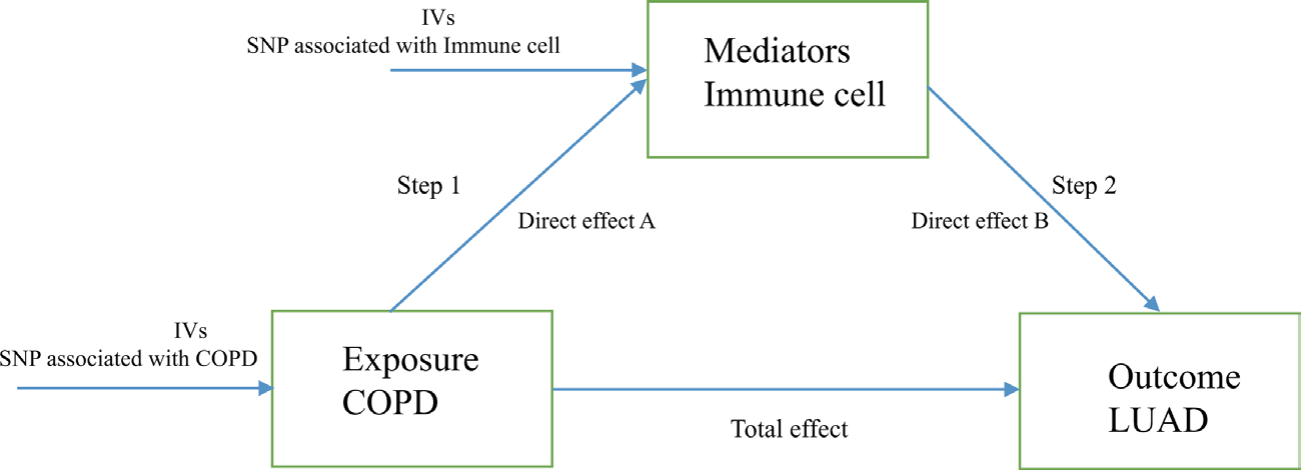
The study design. A 2-sample, 2-step Mendelian randomization (MR) analysis to evaluate the mediate effect of immune cells in the pathway from COPD to LUAD. COPD = chronic obstructive pulmonary disease, LUAD = lung adenocarcinoma, IV = instrumental variable, SNP = single-nucleotide polymorphism.

Using a 2-sample MR approach, we investigated the causal relationships between COPD and LUAD. We then sought to identify and prioritize the causal immune cell types underlying the COPD–LUAD association. Finally, we assessed the mediating role of these immune cells in the pathway from COPD to LUAD. The study design was shown in Figure 1.

### 2.2. Data sources

Genetic variants for all samples were gathered from genome-wide association study (GWAS, https://gwas.mrcieu.ac.uk/). The COPD Cohort (GWAS ID: ebi-a-GCST90018807^[13]^) contained 468 475 samples (13 530 COPD samples and 454 945 control samples) and 24 180 654 SNPs. The LUAD Cohort (TRICL consortium, GWAS ID: ieu-a-984^[14]^), included 11 245 LUAD samples and 65 864 control samples, and 10 345 176 SNPs. 731 immune cell traits (Ebi-a-GCST0001391 to Ebi-a-GCST0002121) contained 6 panels: TBNK (B cells, natural killer cells, T cells), B cells, mature stages of T cells, dendritic cells, monocytes, myeloid cells, and Treg.^[15]^ All participants in the study were from Europe.

### 2.3. Genetic IVs selection

*P* < 5e × 10^−8^ was set as criteria to filter out genetic IVs for COPD and LUAD. According to previous researches,^[16]^ a loose filter condition of SNPs (*P* < 1e × 10^–5^) was applied for determine genetic IVs for 731 immune cell traits. *R*^2^ < 0.001 within ± 10 000 kb distance were set as the threshold of clumping for independence by linkage disequilibrium. Then, we calculated the F-statistics (β/ standard error^2^) and filtrated IVs with *F* statistics > 10.

### 2.4. Statistical analysis

We conducted a 2-sample MR analysis using R packages “TwoSampleMR,” “VariantAnnotation,” and “ieugwasr,” employing 5 methods: “MR Egger,” “Weighted median,” “Inverse variance weighted (IVW),” “Simple mode,” and “Weighted mode.” The primary analysis utilized the random-effects IVW method, which integrates the Wald ratio of individual SNPs, offering robust estimates across diverse scenarios.^[17]^ In cases of heterogeneity, random-effects models replaced fixed-effects models. We assessed heterogeneity using Cochran’s Q statistic and evaluated horizontal pleiotropy with the MR-Egger intercept test.^[18]^ The MR-PRESSO outlier method, implemented via the “MR-PRESSO” R package, corrected horizontal pleiotropy by removing outliers and assessing differences pre- and post-correction.^[19]^ We performed a Leave-one-out analysis to determine if results were influenced by a single SNP. Additionally, the “product of coefficients” method estimated the indirect effect of COPD on LUAD risk mediated by immune cells, with standard errors for indirect effects calculated using the delta method.


$$\begin{array}{l} \beta \left(indirect\;ef\;fect\right)=\beta \left(COPD\;on\;immune\;cells\right)*\beta \left(Immune\;cells\;on\;LUAD\right) \end{array}$$

$$\begin{array}{l} The\;mediated\;proportion=\beta \left(indirect\;ef\;fect\right)/\beta \left(total\;ef\;fect\right) \end{array}$$

## 3. Results

### 3.1. The total effect of COPD on LUAD

11, 14, and 975 SNPs were selected for COPD, LUAD, and 40 immune cell traits (Table S1, Supplemental Digital Content, https://links.lww.com/MD/Q365). The *F* statistics of above SNPs were > 10, demonstrating their suitability as strong instruments.

**Figure 2.**
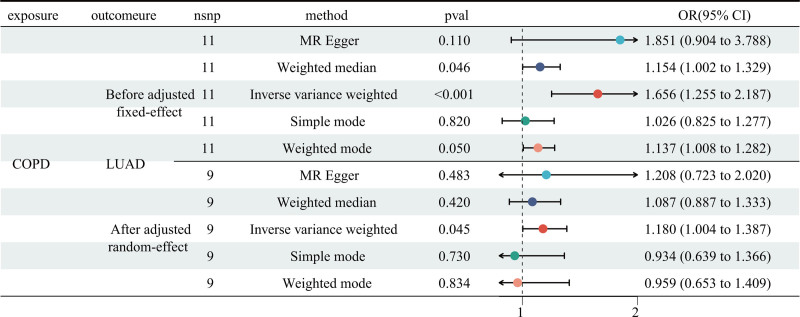
The MR analysis showed the causality of COPD on LUAD. CI = confidence interval, COPD = chronic obstructive pulmonary disease, LUAD = lung adenocarcinoma, MR = Mendelian randomization, OR = odds ratio.

Two-sample MR analysis was employed to estimate the causal effect of COPD on LUAD. The Cochran’s *Q* statistic revealed significant heterogeneity (IVW: *P* < .001; MR-Egger: *P* < .001, Table S2, Supplemental Digital Content, https://links.lww.com/MD/Q365), necessitating the use of a fixed-effect model. The IVW approach demonstrated a robust causal association between COPD and LUAD (odds ratio [OR] = 1.656, 95% confidence interval [CI] [1.252, 2.187], *P* < .001, Fig. 2 and Table S4, Supplemental Digital Content, https://links.lww.com/MD/Q365). Similar results were observed using the weighted median (OR = 1.154, 95% CI [1.002, 1.329], *P* = .046) and weighted mode (OR = 1.137, 95% CI [1.008, 1.282], *P* = .050) methods. However, no significant association was found using the MR-Egger (OR = 1.154, 95% CI [1.002, 1.329], *P* = .110) and simple mode (OR = 1.026, 95% CI [0.825, 1.277], *P* = .820) approaches. The MR-PRESSO distortion test was statistically significant (*P* < .001), although the MR-Egger intercept test did not indicate the presence of horizontal pleiotropy (*P* = .744, Table S3, Supplemental Digital Content, https://links.lww.com/MD/Q365). Then, we corrected the horizontal pleiotropy by removing outliers SNPs (rs3822479; rs9788721). After outlier correction, the random-effect IVW method also demonstrated the causal effect of COPD on LUAD (OR = 1.180, 95% CI [1.004, 1.387], *P* = .045) without heterogeneity (IVW: *P* = .091; MR Egger: *P* = .124; Table S2, Supplemental Digital Content, https://links.lww.com/MD/Q365) and horizontal pleiotropy (MR-PRESSO *P* = .147; Table S3, Supplemental Digital Content, https://links.lww.com/MD/Q365). Further, no single SNP seriously violated COPD’s overall impact on LUAD in the leave-one-out sensitivity analysis.

### 3.2. The effect of COPD on 731 immune cell traits

**Figure 3.**
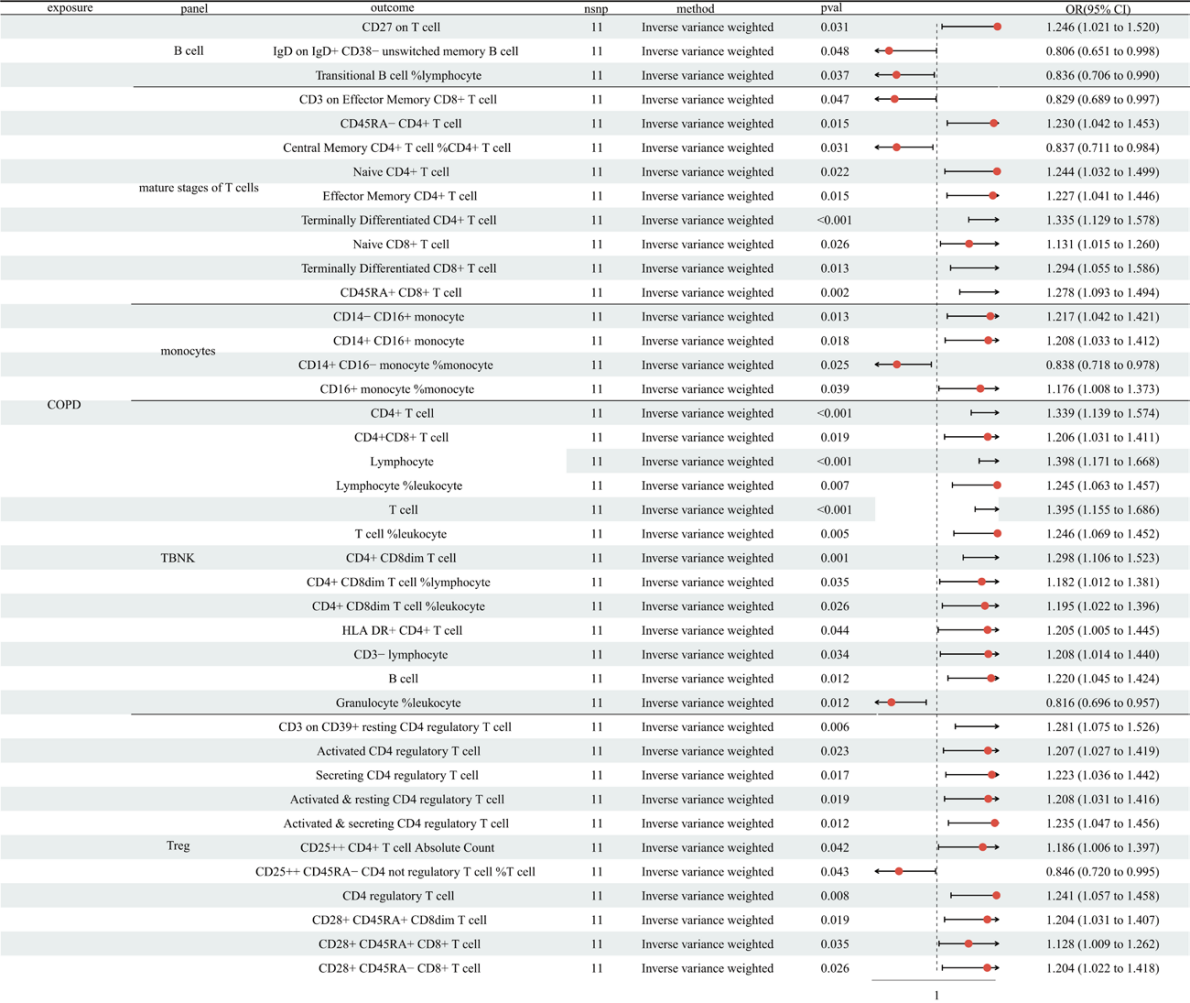
The MR analysis showed COPD was positively related to the level of 33 immune cell traits and negatively related to 7 immune cell traits. COPD = chronic obstructive pulmonary disease, MR = Mendelian randomization

The IVW method showed that COPD was positively correlated with 33 immune cell traits and negatively correlated with 7 immune cell traits (Fig. 3 and Table S4, Supplemental Digital Content, https://links.lww.com/MD/Q365). All *P*-values of Cochran’s *Q* statistic were >.05 (Table S2, Supplemental Digital Content, https://links.lww.com/MD/Q365), indicating undetermined heterogeneity. In addition, the MR Egger intercept test and MR-PRESSO did not show horizontal pleiotropy (Table S3, Supplemental Digital Content, https://links.lww.com/MD/Q365). Furthermore, no SNP significantly violated the overall impact of COPD on immune cell traits.

### 3.3. The effect of immune cell traits on LUAD

**Figure 4.**
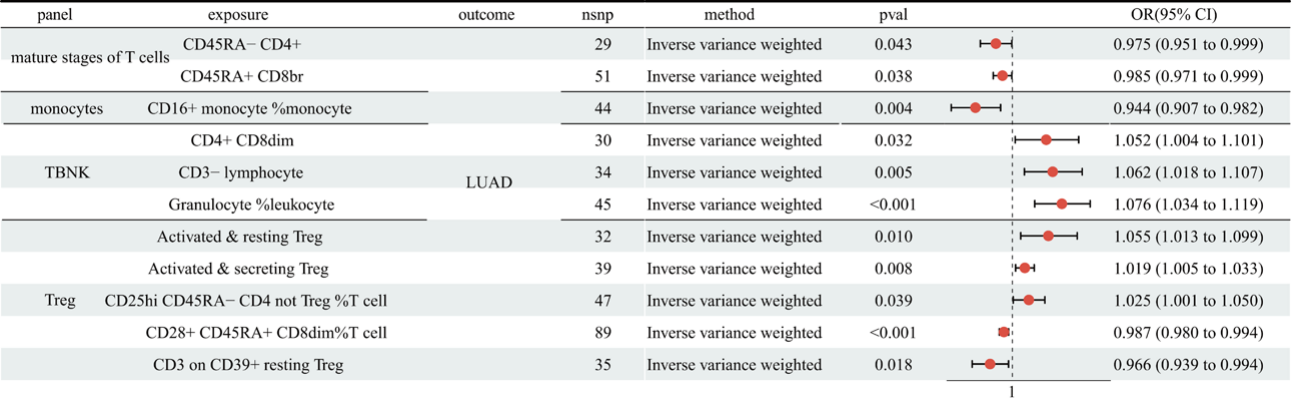
The MR analysis showed 5 immune cell traits had protective effects on LUAD and 6 had adverse effects. LUAD = lung adenocarcinoma, MR = Mendelian randomization

Previously, we detected a remarkable association of COPD with 40 immune cell traits. Then, we analyzed the causal role of 40 immune cell traits on LUAD. The MR analysis illustrated 5 immune cell traits had protective effect on LUAD (CD45RA^−^CD4^+^ T cell: OR = 0.975, 95% CI: 0.951–0.999, *P* = .043; CD45RA^+^ CD8br T cell: OR = 0.985, 95% CI: 0.971–0.999, *P* = .038; CD16^+^ monocyte %monocyte: OR = 0.944, 95% CI: 0.907–0.982, *P* = .004; CD28^+^ CD45RA^+^ CD8dim%T cell: OR = 0.987, 95% CI: 0.980–0.994, *P* < .001; CD3 on CD39^+^ resting Treg: OR = 0.966, 95% CI: 0.939–0.994, *P* = .018; Figure 4 and Table S4, Supplemental Digital Content, https://links.lww.com/MD/Q365). In addition, 6 immune cell traits were related to a higher risk of LUAD (CD4^+^ CD8dim: OR = 1.052, 95% CI: 1.004–1.101, *P* = .032; CD3 − lymphocyte: OR = 1.062, 95% CI: 1.018–1.107, *P* = .005; Granulocyte %leukocyte: OR = 1.076, 95% CI: 1.034–1.119, *P* < .001; Activated & resting Treg: OR = 1.055, 95% CI: 1.013–1.099, *P* = .010; Activated & secreting Treg: OR = 1.019, 95% CI: 1.005–1.033, *P* = .008; CD25hi CD45RA^−^CD4 not Treg %T cell: OR = 1.025, 95% CI: 1.001–1.050, *P* = .039). No heterogeneity and horizontal pleiotropy were observed, and a particular SNP did not drive causal estimates (Table S2; Table S3, Supplemental Digital Content, https://links.lww.com/MD/Q365).

### 3.4. The mediation effect of COPD on LUAD

We identified 11 immune cell traits highly associated with COPD and LUAD. Then, we explored the mediation role of 11 immune cell traits between COPD and LUAD. We found that Activated & resting Treg and Activated & secreting Treg may play a vital role in the pathway from COPD to LUAD. The mediation effect of Activated & resting Treg was 0.010 (95% CI: 0.001–0.021; *P* = .047; Table 1). The proportion of the mediation effect was 6.1%. The mediation effect of Activated & secreting Treg was 0.004 (95% CI: 0.001–0.008; *P* = .044). The proportion of the mediation effect was 2.4%.

**Table 1:** Mediation effect of COPD on LUAD via immune cell traits.

Mediator	Total effect	Direct effect A	Direct effect B	Mediation effect	*P*	Mediated Proportion (%)
	β (95% CI)	β (95% CI)	β (95% CI)	β (95% CI)		
Activated & resting Treg	0.165 (0.083–0.247)	0.189 (0.119 to 0.258)	0.053 (0.035 to 0.072)	0.010 (0.002 to 0.022)	.0469	6.1
Activated & secreting Treg		0.211 (0.132 to 0.289)	0.019 (0.013 to 0.025)	0.004 (0.001 to 0.009)	.0448	2.4
CD25hi CD45RA^-^ CD4 not Treg % T cell		−0.167 (−0.249 to −0.085)	0.025 (0.013 to 0.037)	−0.004 (−0.011 to 0.001)	.148	2.5
CD45RA^-^ CD4^+^		0.207 (0.122 to 0.292)	−0.026 (−0.038 to −0.012)	−0.005 (−0.001 to 0.013)	.118	3.2
CD45RA^+^ CD8br		0.245 (0.165 to 0.325)	−0.015 (−0.026 to −0.007)	−0.004 (−0.008 to −0.001)	.085	2.3
CD16^+^ monocyte % monocyte		0.162 (0.083 to 0.241)	−0.058 (−0.078 to −0.037)	−0.009 (−0.022 to −0.001)	.094	5.7
CD4^+^ CD8dim		0.261 (0.179 to 0.342)	0.050 (0.026 to 0.074)	0.013 (0.001 to 0.030)	.074	8.0
CD3^-^ lymphocyte		0.189 (0.099 to 0.279)	0.060 (0.038 to 0.080)	0.011 (0.001 to 0.027)	.090	6.8
Granulocyte % leukocyte		−0.204 (−0.285 to −0.122)	0.073 (0.052 to 0.092)	−0.015 (−0.031 to −0.003)	.059	9.0
CD28^+^ CD45RA^+^ CD8dim		0.186 (0.106 to 0.265)	−0.013 (−0.016 to −0.009)	−0.002 (−0.005 to −0.001)	.051	1.5
CD3 on CD39^+^ resting		0.247 (0.157 to 0.337)	−0.035 (−0.049 to −0.020)	−0.009 (−0.019 to −0.001)	.072	5.2

Total effect: The causal role of COPD on LUAD. Direct effect A: The causal role of COPD on immune cell traits. Direct effect B: The causal role of immune cell traits on LUAD.

CI = confidence interval, COPD = chronic obstructive pulmonary disease, LUAD = lung adenocarcinoma.

### 3.5. A reverse MR analysis

**Figure 5.**
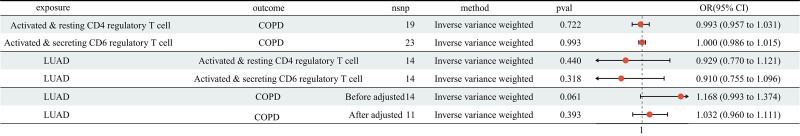
A reverse MR analysis showed no causal role of LUAD on COPD, Activated & resting Treg and Activated & secreting Treg cells, as well as no causal role of Activated & resting Treg and Activated & secreting Treg cells on COPD. COPD = chronic obstructive pulmonary disease, LUAD = lung adenocarcinoma, MR = Mendelian randomization

Finally, we performed a reverse MR analysis to analyze the association of genetically predicted LUAD with COPD and immune cell traits, as well as immune cell traits with COPD. We did not detect statistically significant causal effect of LUAD on COPD (OR = 1.168, 95% CI: 0.993–1.374, *P* = .061), Activated & resting Treg (OR = 1.929, 95% CI: 0.770–1.121, *P* = .440), and Activated & secreting Treg (OR = 0.910, 95% CI: 0.755–1.096, *P* = .318) by IVW method (Fig. 5). Similar results were observed in the causal effect of Activated & resting Treg (OR = 0.993, 95% CI: 0.957–1.031, *P* = .722) and Activated & secreting Treg (OR = 1.000, 95% CI: 0.986–1.015, *P* = .993) on COPD. No heterogeneity was detected except in the analysis of the causal role of LUAD on COPD (IVW: *P* < .001; MR Egger: *P* < .001; Table S2, Supplemental Digital Content, https://links.lww.com/MD/Q365). In the analysis of the causal role of LUAD on COPD, the MR-Egger intercept test did not show horizontal pleiotropy (*P* = .735; Table S3, Supplemental Digital Content, https://links.lww.com/MD/Q365). However, the MR-PRESSO distortion test was statistically significant (*P* < .001; Table S3). We removed the outliers SNPs (rs13314271, rs55781567, rs71658797) to adjust the horizontal pleiotropy. After outlier correction, we performed MR analysis again, and the IVW method indicated similar results (OR = 1.032, 95% CI: 0.960–1.111, *P* = .9393) and no heterogeneity and horizontal pleiotropy (Table S2; Table S3, Supplemental Digital Content, https://links.lww.com/MD/Q365).

## 4. Discussion

COPD appears to increase the likelihood of developing LC compared to individuals without COPD, suggesting that COPD may serve as an independent risk factor for LC.^[4]^ Furthermore, the progression from COPD to LUAD is often associated with a poor prognosis.^[3]^ In this study, we examined the correlation between genetically predicted COPD and the genetic susceptibility to LUAD using a 2-sample MR analysis. The MR analysis revealed a strong causal relationship between COPD and LUAD, with an odds ratio (OR) of 1.656. After accounting for horizontal pleiotropy, the OR decreased to 1.180, still indicating that COPD elevates the risk of LUAD. This finding aligns with a case-control study result (OR: 1.29, 95% CI: 1.00–1.68).^[20]^

It is well known that COPD promotes tumor progression and reduces the efficacy of LC therapy.^[21]^ However, there is limited research to clarify the complex disease mechanism connecting COPD and LC. Chronic airway inflammation and infiltration of immune cells are critical characteristics of COPD, and subsequently remodel the bronchus, resulting in the development of COPD.^[22]^ In addition, a study investigated the expression of peripheral blood expression immune cells in COPD patients and revealed the expression of several immune cells increased, such as CD3^+^ CD8^+^ T cells.^[23]^ In the study, we applied a 2-sample MR analysis to analyze the causal role of COPD on 731 immune cell traits. The results illustrated COPD was related to the level of 40 immune cell traits, including 3 traits in the B cell panel, 9 traits in the mature stages of the T cells panel, 4 traits in the monocytes panel, 13 traits in the TBNK panel, and 11 in the Treg panel.

Immune cell infiltration into tumor tissue has been implicated in tumor metastasis, drug resistance, and immune evasion.^[24]^ Previous work identified the causal effect of COPD on 40 immune cell traits. Here, we explored the association of those 40 immune cell traits with LUAD. MR analysis revealed a clear causal relationship between 11 immune cell traits and LUAD. Specifically, 2 of these traits were in the mature T cell panel, 1 was in the monocyte panel, 3 were in the TBNK panel, and 5 were in the regulatory T cell (Treg) panel. Finally, we evaluated the mediating effect of these 11 immune cell traits in the pathway from COPD to LUAD. Mediation analyses showed that the mediating effects of activated & resting Treg and activated & secreting Treg were 0.010 and 0.004, respectively, accounting for 6.1% and 2.4% of the total effect. This suggests that activated & resting Treg and activated & secreting Treg may be potential mediators in the relationship between LUAD risk and COPD. These findings provide genetic evidence for the connection between COPD and LUAD, and elucidate the underlying mechanisms. Regulatory T (Treg) cells were a subgroup of CD4^+^lymphocytes that restrained the proliferation of other T cells and secreted anti-inflammatory cytokines, like IL-10 and TGF-β, providing the body with the necessary protection against over-activated immune responses by killing effector cells or APCs via releasing granzyme A and perforin.^[25,26]^ Previous literature has reported in COPD patients, especially patients with long-term smoking, the level of Treg cells increased,^[27]^ which was in line with our finding. High levels of Treg cells contribute to the control of lung inflammation, maintaining local immune homeostasis and ultimately inhibiting the development of COPD.^[28]^ Herein, we found that Activated & resting Treg and Activated & secreting Treg increased the risk of LUAD, which was confirmed by previous research: Treg cells promoted the progression and metastasis of LUAD by inhibiting CD8 + T cell-mediated anti-tumor immunity. Unlike its role in COPD, the role of Treg cells in cancer immunity is disadvantaged factor due to Treg cells recruited in tumor tissues could contribute to tumor immune escape.^[29,30]^

This study provided us with a good understanding of the relationship between COPD and LUAD, and the mediation effect of Activated & resting Treg and Activated & secreting Treg in the pathway from COPD to LUAD, which presented a potential strategy for preventing LUAD and a mean for treating LUAD patients with COPD Nevertheless, some limitations still need to be addressed. The data were obtained from public databases. To avoid potential pleiotropy, we identified and removed potential confounding SNPs. Additionally, we conducted leave-one-out analysis and the MR-PRESSO outlier test to assess horizontal pleiotropy. However, we cannot exclude the possibility of residual confounding or heterogeneity. The study population was limited to individuals of European ancestry, which may restrict the generalizability of the findings. The results suggest that the pathway from COPD to LUAD is partially mediated by activated and resting regulatory T cells (Tregs), as well as activated and secreted Tregs. However, the mediation effects were modest, accounting for only 6.1% and 2.4% of the total effect, respectively. Other factors may also contribute to this pathway. Larger prospective studies and further basic research are needed to better elucidate the role of Treg subsets in the COPD-LUAD relationship.

## 5. Conclusion

The study showed the causal role of COPD on LUAD and the relationship may be partial mediated by Activated & resting Treg cells and Activated & secreting Treg cells, which was needed further clinical and basic experiments to validate.

## Acknowledgments

The authors thank all the investigators and participants for sharing the GWAS data.

## Author contributions

**Data curation:** Xinyu Liu, Jing Xu.

**Conceptualization:** Sheng Wang.

**Formal analysis:** Sheng Wang.

**Methodology:** Xinyu Liu, Jing Xu, Sheng Wang.

**Project administration:** Sheng Wang.

**Software:** Jing Xu.

**Validation:** Xinyu Liu, Jing Xu.

**Writing – original draft:** Xinyu Liu, Jing Xu.

**Writing – review & editing:** Sheng Wang.

## Supplementary Material


